# Supplementary material for: Nonlinear cavity optomechanics with nanomechanical thermal fluctuations
Source: Nat Commun. 2017 Jul 7;8:ncomms16024. doi: 10.1038/ncomms16024 (PMC5504350; doi:10.1038/ncomms16024)
Supplement: Supplementary Information [file ncomms16024-s1.pdf]

File name: Supplementary Information
Description: Supplementary Figures, Supplementary Notes and Supplementary References

File name: Peer Review File
Description:

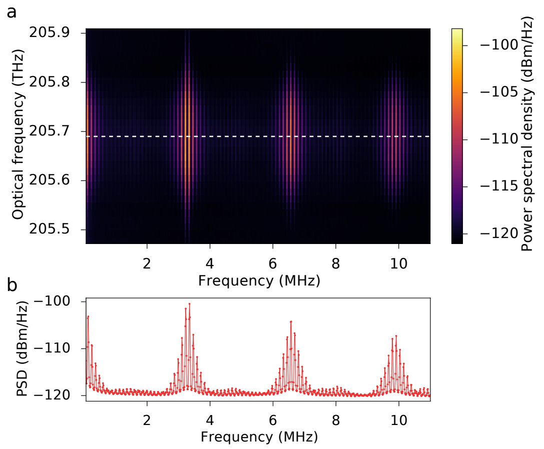

Supplementary Figure 1 | Nonlinear transduction as a function of detuning. (a) Electronic spectrum analyser signal taken at room temperature, as a function of the optical frequency. (b) Cross section at cavity resonance frequency corresponding to Fig. 3a in the main text. As can be seen in (a), due to the quadrature-averaged homodyne detection, all peaks show a single-peaked detuning dependence which is maximum at resonance.

Supplementary Note 1
Power dependence of transduced mechanical motion

The transduction of mechanical motion depends on the optical power used, as shown in the dependence of $\langle P^2 \rangle$ on $A^2 \propto P_{\text{in}} P_{\text{LO}}$ in Eq. 2 of the main text. In particular, we expect the measured band power to be linear in both the power in the signal arm P_{in}, incident on the cavity, and the power in the reference arm P_{LO}. Deviations from a linear relation indicate heating or cooling of the mechanical motion due to the additional laser power [1]. Supplementary Figure 2

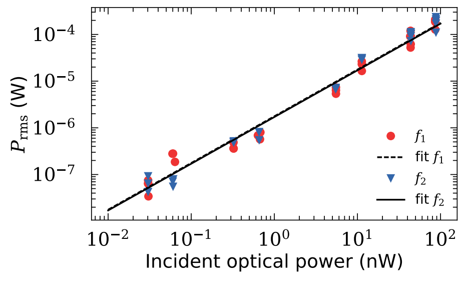

Supplementary Figure 2 | Power dependence. Measured band power of the two fundamental mechanical frequencies f_1 and f_2 (area under the fitted Lorentzian peak) as a function of the incident optical power P_{in}, with the laser frequency on-resonance with the cavity. The power in the reference arm, P_{LO}, was kept constant. The black dashed and solid lines show linear fits to the data for f_1 and f_2, respectively.

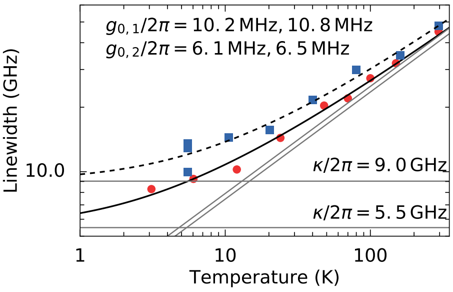

Supplementary Figure 3 | Characterisation of a second device. Optical linewidth versus temperature data for a second device. For the dataset with blue squares a second cryostat window at 30 K was not in place, hence exposing the sample to thermal radiation from the outer window at room temperature. The solid and dashed black lines are fits with a model that assumes a constant Lorentzian intrinsic linewidth κ convolved with a Gaussian with a width L_G that depends on $\sqrt{T}$. The asymptotes (straight grey lines) of the fit functions allow us to extract κ and the optomechanical coupling rates $g_{0,i}$ for the two mechanical modes ($i = 1, 2$). Extracted values are shown in the figure. We assign the discrepancy in extracted values of κ to bad thermalisation of the sample in the case where the inner window was not in place.

shows transduced mechanical motion as a function of the incident optical power, with the laser on-resonance with the cavity and while the device was cooled to 3 K. The data was corrected for the local oscillator power, which was mostly kept constant and did not vary by more than a factor 10. At higher powers (near 100 nW), fluctuations in the centre frequency and shape of the peak could be observed (similar to the effects shown off-resonance in Fig. 5 in the main text), however the peak area still follows the linear dependence as a function of the optical power incident at the structure. This shows that for all probe powers used here, there is no significant heating due to the incident laser light.

Supplementary Note 2
Characterisation of a second device and estimation of thermalisation

The data labelled as "device 2" were taken on a sample with a slightly different design, which did not include the double-period modulation to increase the outcoupling, as well as a different shape of the holes. The optical linewidth as a function of temperature for this device is shown in Supplementary Figure 3, in two separate data sets. The data set shown by blue squares was taken with only one window in the cryostat, which was at room temperature, such that the sample was not shielded from thermal radiation. The other dataset was taken with the second window (cooled to around 50 K) in place. Therefore the sample temperature for the blue dataset was most likely higher than the thermometer temperature, which we used to plot and fit these data. Although this leads to a relatively large difference in the fitted optical loss rate κ, both datasets yield very similar coupling rates g_0, since at high temperatures the sample is well thermalised. We note that the different estimate of κ in the absence of

the cold window is of course the direct result of the fact that the nanobeam temperature is larger than that of the nearby thermometer (which is plotted on the horizontal axis of Supplementary Figure 3). Comparing the solid and dashed curves shows that the temperature is raised by ~10 degrees from the base temperature. Since this temperature rise can be related to the presence of the nearby 300 K window, we can now estimate the rise that is expected due to the ~ 50 K cold window that is in place during the measurements reported in the main text. Assuming a $\propto T^4$ dependence of heat transfer rates results in an expected temperature rise of ~ 0.1 K. This shows that the nanobeams are very likely thermalised well down to the lowest cryostat temperatures. We finally remark that it is not straightforward in our case to verify thermalisation using a phase modulation calibration, as described by Gorodetsky and coworkers[2]: The strong intrinsic cavity frequency modulation means that such a calibration tone will inevitably mix with the mechanically-induced modulations, such that its magnitude is strongly altered and in fact itself depends on temperature.

Supplementary Note 3
Quadratic displacement measurements

The signal-to-noise ratio (SNR) of a quadratic displacement measurement, in the regime where the order-by-order approximation is valid, can be found by considering Eq. 2 in the main text, and assuming the measurement is shot-noise limited. If the optical power in the reference arm is much larger than in the signal arm of the interferometer ($P_{\mathrm{LO}} \gg P_{\mathrm{in}}$), the power spectral density due to the optical shot noise is given by $S_{PP}^{\mathrm{SN}} = \hbar\omega_{\mathrm{c}} P_{\mathrm{LO}} h'$, with h' quantifying the measurement efficiency for the local oscillator light (that is, losses between the homodyne beamsplitter and the detector, and the quantum efficiency of the detector). The band power at $2\Omega_{\mathrm{m}}$ due to thermal mechanical fluctuations is given by Eq. 2 in the main text, with $k = 2$ and $\langle \delta\omega^2 \rangle = 2\bar{n}_{\mathrm{th}} g_0^2$, leading to

$$\langle P^2 \rangle_{2\Omega_{\mathrm{m}}} = 512 P_{\mathrm{in}} P_{\mathrm{LO}} h \eta^2 \left(\frac{g_0}{\kappa}\right)^4 \bar{n}_{\mathrm{th}}^2, \tag{1}$$

where we also substituted $A^2 = 8 P_{\mathrm{in}} P_{\mathrm{LO}} h \eta^2$, where h accounts for the finite measurement efficiency for the signal beam (Supplementary Note 4). The power spectral density will show a Lorentzian peak with linewidth 2Γ at the frequency $2\Omega_{\mathrm{m}}$, which means its peak value is related to the band power (area under the peak) as $S_{PP}^{\max} = \langle P^2 \rangle_{2\Omega_{\mathrm{m}}} / 2\Gamma$. Finally, we take the signal-to-noise ratio

$$\mathrm{SNR} \equiv \frac{S_{PP}^{\max}}{S_{PP}^{\mathrm{SN}}} = 256 \frac{P_{\mathrm{in}} \xi \eta^2}{\hbar\omega_{\mathrm{c}} \Gamma} \left(\frac{g_0}{\kappa}\right)^4 \bar{n}_{\mathrm{th}}^2, \tag{2}$$

where we have defined $\xi \equiv h/h'$. We set SNR to 1 to find the imprecision in terms of a phonon number n_{imp}, leading to

$$\frac{1}{n_{\mathrm{imp}}} = 16 \left(\frac{g_0}{\kappa}\right)^2 \eta \sqrt{\frac{P_{\mathrm{in}} \xi}{\hbar\omega_{\mathrm{c}} \Gamma}}. \tag{3}$$

Supplementary Figure 4 shows additional data for the quadratic measurement presented in the main text. The top panel shows the peak around the

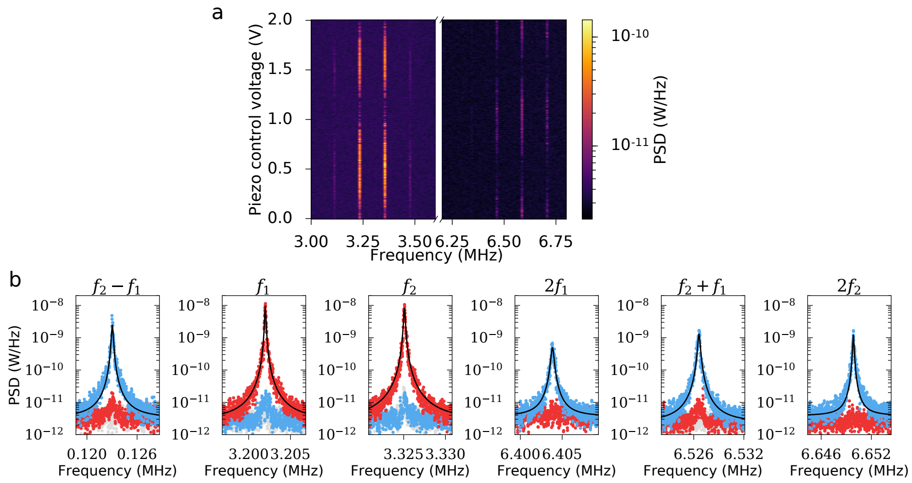

Supplementary Figure 4 | Selective linear and quadratic measurement. (a) Spectra showing the first and second order transduction obtained simultaneously, as a function of the piezo mirror position. (b) Maximum and minimum linear and quadratic transduction at different settings of the homodyne phase, for the fundamental frequencies f_1 and f_2 as well as all second-order peaks. Red (blue) data points: homodyne phase set to maximize linear (quadratic) transduction; black solid lines show Lorentzian fits to the peaks. Grey data points were taken with no incident optical power in the signal arm. For these measurements, the sample was at 3 K, and incident optical power was 12.8 nW.

fundamental frequency decrease at the same piezo mirror position where the peaks at twice the frequency are strongest, further confirming the ability to suppress the linear measurement while performing the quadratic measurement. The bottom panel shows the linear and quadratic measurements at optimum positions of the piezo mirror for both fundamental frequencies and all 4 second-order combinations.

Supplementary Note 4
Coupling and measurement efficiency

Supplementary Figure 5 shows the fitted centre frequency of the mechanical resonance f_1 at 3 K with $P_{\text{in}} = 20.6\,\text{nW}$, corresponding to the spectrogram shown in Fig. 5a in the main text. Fitting the change in frequency as a function of the optical detuning with the function describing the optical spring effect in the bad-cavity limit[3;1] provides us with an estimate of the incoupling efficiency η. We measure the optical power P_{in} before the objective lens, so aside from polarisation mismatch and mode overlap with a Gaussian beam, η also includes transmission losses from the (anti-reflection coated) objective lens and 2 (uncoated) cryostat windows. The average over 5 measurements at different input power yielded a coupling efficiency $\eta = 1.3\,\%$, with a standard deviation of $0.3\,\%$.

This analysis ignores the effects of the large thermal fluctuations, which as

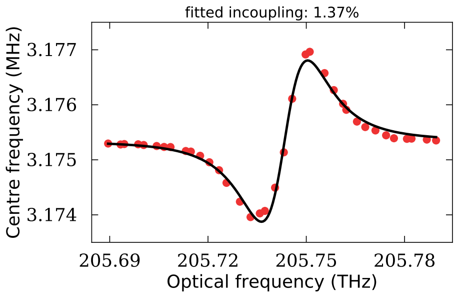

Supplementary Figure 5 | Optical spring effect at low temperature. Fitted centre frequency of the mechanical resonance f_1 with the structure at 3 K and $P_{\text{in}} =$ 20.6 nW, as a function of the laser frequency (corresponding to the data set shown in Fig. 5a in the main text). The solid line shows a fit of the data according to the linearised model for the optical spring effect, where the magnitude of the effect depends on the incoupling efficiency η. The extracted value is shown in the figure.

we show in the main text broadens and reduces the strength of the optical spring effect. This means the obtained coupling efficiency provides a lower limit for the actual coupling efficiency. However, the numerical model we used to generate the simulated spectra in Fig. 5 in the main text shows that the fluctuations at 3 K mainly broaden the mechanical spectra but do not yet lead to strong reduction in the strength of the optical spring effect.

In order to characterise the full measurement efficiency $\zeta = h\eta$, we also need to consider the quantum efficiency of our homodyne detection (h). This includes mainly the overlap of the signal and local oscillator beams ($\sim 40\,\%$ in the measurements presented), and the quantum efficiency of our photodiode ($\sim 80\,\%$). We also lose some photons via losses in the optical path and by using a variable aperture to balance the powers reaching the two photodiodes of our balanced photodetector. Overall, we estimate $h \approx 25\,\%$ in experiments in this paper. We note that we did not try extensively to optimise h, and we expect it could be improved significantly with modest effort. This would be essential for the feedback schemes mentioned in the main text.

Supplementary Note 5
Radiation pressure force in a cavity with large thermal fluctuations

We express the radiation pressure force in the cavity as

$$F_{\text{rad}} = \frac{\hbar(\partial\omega_{\text{c}}/\partial x)n_{\text{c}}^{\text{max}}}{1+u^2}, \tag{4}$$

where $u \equiv \frac{2}{\kappa}(\overline{\Delta} + (\partial\omega_{\text{c}}/\partial x)x)$ and $n_{\text{c}}^{\text{max}}$ is the maximum number of photons in the cavity when it is driven at resonance. We assume the resonator moves harmonically: $x(t) = x_0 \sin\Omega t$. This results in a time-dependent normalized detuning $u(t) = \overline{u}(\overline{\Delta}) + u_0 \sin\Omega t$, where $u_0 = 2(\partial\omega_{\text{c}}/\partial x)x_0/\kappa$.

Given an amplitude x_0, we extract the first Fourier coefficient of the force

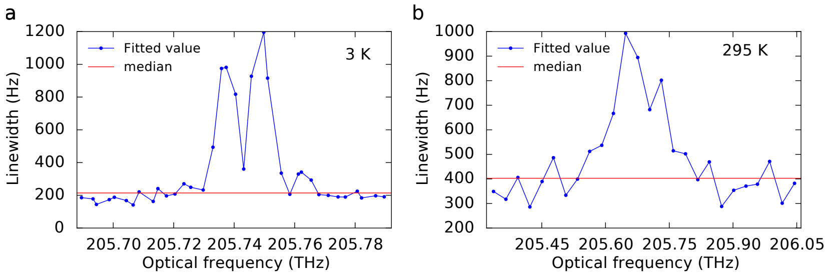

Supplementary Figure 6 | Mechanical linewidth. Fitted mechanical linewidth as a function of laser frequency at (a) 3 K and (b) 295 K, corresponding to the detuning sweeps shown in the main text in Fig. 5a & d. The solid horizontal lines show the median value of the fitted linewidth, which was used as the intrinsic linewidth for the simulated spectrograms shown in Fig. 5b & e in the main text.

(at the resulting harmonic frequency Ω):

$$a_1^{\mathrm{rad}} = \frac{\Omega}{\pi} \int_0^{2\pi/\Omega} F_{\mathrm{rad}}(t') \sin \Omega t' dt'. \tag{5}$$

In analogy with the mechanical restoring force, we can calculate an effective spring constant from the Fourier coefficient: with $F = -kx$ and $x(t) = x_0 \sin \Omega t$, $F = \sum_{n=1}^{\infty} a_n \sin n\Omega t$ implies that $a_1 = -kx_0$.

Finally, a correction on the spring constant results in a correction on the frequency Ω:

$$\Omega = \sqrt{\frac{k + k_{\mathrm{rad}}}{m}} = \sqrt{\frac{k}{m}} \sqrt{1 + \frac{k_{\mathrm{rad}}}{k}}, \tag{6}$$

which we can approximate using $\sqrt{1+x} \approx 1 + x/2 + \ldots$:

$$\Omega \approx \sqrt{\frac{k}{m}} (1 + \frac{k_{\mathrm{rad}}}{2k}), \tag{7}$$

therefore

$$\delta\Omega \approx \sqrt{\frac{k}{m}} \frac{k_{\mathrm{rad}}}{2k} = \frac{k_{\mathrm{rad}}}{2m\Omega}. \tag{8}$$

To obtain the results presented in Fig. 5 in the main text, we perform this calculation for 10^4 amplitudes x_0. These amplitudes are sampled from a Rayleigh distribution, which describes the thermal motion (equivalently, we take $\sqrt{x_1^2 + x_2^2}$ with x_1, x_2 independently sampled from a normal distribution). The mean amplitude is set such that the variance of u corresponds to the total variance we expect from both mechanical modes, using the measured values of $\kappa, g_{0,i}, \Omega_{\mathrm{m},i}$ and the temperature. To generate spectra that can be compared to the experimental data, we then average together Lorentzian lineshapes with centre frequencies given by the resulting set of frequency shifts. Since the mechanical linewidth changes with temperature, we choose the widths of these individual Lorentzian peaks based on the median value of the measured linewidth.

As Supplementary Figure 6 shows, this is a good approximation of the intrinsic mechanical linewidth, which we measure for large detuning.

Supplementary References

[1] Aspelmeyer, M., Kippenberg, T. J. & Marquardt, F. Cavity optomechanics. *Rev. Mod. Phys.* **86**, 1391–1452 (2014).

[2] Gorodetsky, M. L., Schliesser, A., Anetsberger, G., Deleglise, S. & Kippenberg, T. J. Determination of the vacuum optomechanical coupling rate using frequency noise calibration. *Opt. Express* **18**, 23236–46 (2010).

[3] Leijssen, R. & Verhagen, E. Strong optomechanical interactions in a sliced photonic crystal nanobeam. *Sci. Rep.* **5**, 15974 (2015).
